# Physiology

**Published:** 1911-07-15

**Authors:** 


PHYSIOLOGY.—This is a brief text manual of a course of lectures delivered to the dental students at the University of Michigan, by Professor Carl J. Wiggers, and published by George Wahr, Ann Arbor, Mich. The selection of the material has been made with the idea in mind to present, not dental physiology, but the fundamental facts of general physiology which are clearly established and which will have material bearing on the collateral studies of the dental curriculum. The treatment, while selective and somewhat abridged, is thoroughly scientific and modern. It will serve excellently as a book of review and for courses in schools and colleges where physiological facts and principles only can be presented, because of lack of time. While all established facts are amply stated, little space is given to a discussion of unsettled and debatable theories. It is a good book for a dentist to have in his library, as he can rehearse his physiological knowledge at his pleasure and without the tedious and fatiguing effort of the usual treatise on physiology. The book is made more valuable by such illustration as will shorten the text and make it more clear, and a synoptic summary of each subject discussed is printed at the close of its consideration.

